# Utility-weighted modified rankin scale scores in patients with ischemic stroke: a multicenter observational study

**DOI:** 10.1007/s11136-025-04114-7

**Published:** 2026-01-09

**Authors:** Fumi Irie, Koutarou Matsumoto, Ryu Matsuo, Yoshinobu Wakisaka, Tetsuro Ago, Takanari Kitazono, Masahiro Kamouchi, onbehalf of the Fukuoka Stroke Registry Investigators

**Affiliations:** 1 Department of Health Care Administration and Management, Graduate School of Medical Sciences, Kyushu University, 3-1-1 Maidashi, Higashi-Ku, Fukuoka, 812-8582 Japan; 2 Department of Medicine and Clinical Science, Graduate School of Medical Sciences, Kyushu University, Fukuoka, Japan; 3 Center for Cohort Studies, Graduate School of Medical Sciences, Kyushu University, Fukuoka, Japan

**Keywords:** Quality of life, Stroke, Activities of daily living, Utility value

## Abstract

**Purpose:**

This study assessed patient-reported health-related quality of life (QOL) in a real-world cohort of patients with ischemic stroke and estimated utility-weighted modified Rankin Scale (UW-mRS) scores.

**Methods:**

We included 1452 patients with ischemic stroke (median age: 75 [67–83] years; 41.0% female) from a multicenter hospital-based stroke registry in Japan. QOL was evaluated using the EQ-5D-5L with Japanese-specific utility values. Both EQ-5D utility values and mRS scores were assessed after completion of acute treatment. UW-mRS scores were estimated using ordinary least squares (OLS) and Tobit regression models.

**Results:**

The mean (SD) EQ-5D utility value was 0.68 (0.27). Higher mRS scores were associated with greater severity and frequency of problems across EQ-5D domains, with a marked decline observed between mRS scores 3 and 4. UW-mRS scores estimated by OLS were: mRS 0: 0.90, mRS 1: 0.85, mRS 2: 0.74, mRS 3: 0.62, mRS 4: 0.41, and mRS 5: 0.28. The Tobit model yielded slightly higher values for mRS 1–3. UW-mRS estimates remained largely consistent across other regression models (fractional logit/probit, beta regression, and two-part models). However, UW-mRS values differed between early (≤ 14 days from onset) and late (> 14 days) assessments and varied considerably when utility values were calculated using tariffs from other countries or with the EQ-5D-3L crosswalk.

**Conclusion:**

In this real-world, consecutive cohort of hospitalized Japanese patients with ischemic stroke, utility values were estimated using the EQ-5D-5L, and the UW-mRS was characterized as a practical tool for deriving utility values from mRS scores.

**Supplementary Information:**

The online version contains supplementary material available at 10.1007/s11136-025-04114-7.

## Abstract (plain language summary)

Stroke can have a profound impact on individual’s lives, limiting physical abilities and adversely affecting overall well-being. In this study, we asked hospitalized patients with ischemic stroke in routine clinical practice to report their health status using the EQ-5D-5L, a questionnaire that measures health-related quality of life (HRQoL) across five domains: mobility, self-care, usual activities, pain/discomfort, and anxiety/depression. We estimated utility values for these patients and mapped scores on the modified Rankin Scale (mRS), a widely used measure of post-stroke functional status, to EQ-5D utility values. Our findings demonstrate a strong inverse relationship between functional disability and HRQoL, with the steepest decline observed between moderate and severe disability (mRS scores 3 to 4). The utility-weighted mRS (UW-mRS) scores derived from this real-world cohort may provide a practical approach for estimating patient-reported HRQoL from mRS scores commonly used in stroke research. However, UW-mRS values varied depending on the time from stroke onset to assessment, the value sets applied from different countries, and whether EQ-5D-5L results were mapped to the 3L. Therefore, caution is warranted when applying the UW-mRS for mapping across different settings or populations.

**Supplementary Information:**

The online version contains supplementary material available at 10.1007/s11136-025-04114-7.

## Introduction

Stroke outcomes have traditionally been evaluated using objective indicators such as neurological severity, functional status, and survival prognosis—primarily assessed by clinicians. Among these, the modified Rankin Scale (mRS) is a widely used measure of functional outcomes following stroke [1–3]. However, in the context of patient-centered care, it is essential to assess stroke outcomes from the patient’s perspective [4, 5]. Health-related quality of life (QOL) has increasingly been recognized as a crucial subjective outcome measure in stroke care [6, 7].

The EQ-5D is a questionnaire developed by the EuroQoL Group to measure health-related QOL [8, 9]. It is applicable across a broad range of diseases, including stroke, and has been adopted worldwide [10–13]. Each health state is represented by a code derived from the five dimensions and levels, and utility values are calculated by applying this code to country-specific value sets (tariffs). These utility values quantify and standardize patients’ QOL according to societal preferences and are primarily used in health economic evaluations. However, EQ-5D data are not always collected in clinical studies; therefore, efforts have been made to convert clinical outcomes into EQ-5D utility values [14]. In particular, the mRS is the gold-standard clinical indicator of stroke outcomes. Consequently, mRS scores have been mapped to EQ-5D utility values, leading to the development of the utility-weighted mRS (UW-mRS) [15–22].

Most studies investigating the UW-mRS have relied on clinical trial data, and only a few have examined patients in routine clinical practice or general populations [23]. This raises concerns about generalizability, as trial populations are typically younger and more functionally independent prior to stroke onset than those encountered in real-world settings [1, 15–22]. Furthermore, prior research has been conducted predominantly in Europe and North America, with limited data available from other regions [17–19]. Given that UW-mRS scores may vary depending on healthcare systems and the availability of social support [18, 21], further research in diverse social and healthcare settings is warranted.

In this context, we conducted a prospective observational study using data from the Fukuoka Stroke Registry (FSR) in Fukuoka, Japan, to investigate clinical outcomes reported by patients with ischemic stroke. The objectives of this study were to evaluate the QOL of consecutively admitted patients with stroke in a real-world setting using the EQ-5D-5L as a patient-reported outcome measure, estimate utility values derived from the EQ-5D-5L, and derive conversion values to map mRS scores to EQ-5D utility values.

## Methods

### Study design and setting

This study used data from the FSR (UMIN Clinical Trial Registry: 000000800), a multicenter, hospital-based stroke registry that included patients admitted to seven stroke centers in Fukuoka, Japan (Online Resource 1: Appendix). This was a sub-study of the FSR focused on assessing the QOL of patients with stroke using the EQ-5D-5L. The study protocol was approved by the ethics committees of all participating institutions (Ethics approval numbers: Kyushu University Institutional Review Board for Clinical Research: 22,086–01; Kyushu Medical Center Institutional Review Board: R06-03; Clinical Research Review Board of Fukuokahigashi Medical Center: 29-C-38; Fukuoka Red Cross Hospital Institutional Review Board: 629; St. Mary’s Hospital Research Ethics Review Committee: S13-0110; Steel Memorial Yawata Hospital Ethics Committee: 06–04-13; Kyushu Rosai Hospital Institutional Review Board: 21–8). Written informed consent was obtained from all patients or, when necessary, from close family members.

We included consecutive patients with ischemic stroke who were admitted within 7 days of stroke onset and had symptoms persisting for more than 24 h between April 2018 and September 2019. Of 1695 patients admitted during the study period, 23 who died during hospitalization were excluded. Of the remaining 1,672 patients, 1505 provided consent and completed the EQ-5D-5L. After excluding 53 patients due to incomplete EQ-5D data, a final cohort of 1,452 patients was included in the analysis.

### QOL and mRS measurement

QOL was assessed using the Japanese version of the EQ-5D-5L (©EuroQol Research Foundation, EQ-5D™ is a trademark of the EuroQol Research Foundation). The EQ-5D utility values were calculated based on patient responses to five domains: mobility, self-care, usual activities, pain/discomfort, and anxiety/depression. Each domain was rated on a five-point scale: no problem, mild, moderate, severe, or extreme problem. These responses were converted into EQ-5D utility values ranging from -0.025 to 1 using a Japanese-specific tariff [24, 25]. EQ-5D responses were collected after symptom stabilization following acute stroke treatment. QOL was assessed at a mean (SD) of 15.0 (9.6) days after stroke onset. Given the variation in time from stroke onset to assessment, evaluations were classified as early (≤ 14 days) or late (> 14 days). When patients were unable to respond, a proxy respondent, typically a family member, completed the questionnaire.

Functional status was assessed using the mRS at discharge, after completion of acute stroke care and stabilization of the patients’ conditions. The mRS was assessed at a mean (SD) of 20.8 (11.4) days after stroke onset. The mRS is a widely used scale for evaluating functional independence after stroke, ranging from 0 (no symptoms) to 5 (severe dependence), with 6 indicating death, yielding a total of seven grades [26]. Stroke specialists, blinded to the EQ-5D results, performed the mRS evaluations, which were subsequently reviewed by attending physicians who were members of the FSR steering committee.

### Variables

To explore factors related to QOL, data on patient demographics and clinical characteristics were collected, including age, sex, comorbidities, and risk factors (hypertension, diabetes mellitus, dyslipidemia, atrial fibrillation, smoking history, alcohol consumption history, and body mass index [BMI]), as well as stroke history, pre-stroke functional dependency, ischemic stroke subtype, and neurological severity as assessed using the National Institutes of Health Stroke Scale. Definitions of these variables are provided in the Supplemental Methods.

### Statistical analysis

Background characteristics and EQ-5D utility values were compared across mRS groups using the Kruskal–Wallis test or the χ^2^ test. Trends across mRS categories were assessed using the Cochran-Armitage trend test or the Jonckheere-Terpstra trend test. Spearman’s rank correlation coefficient was used to evaluate the correlation between mRS scores and EQ-5D utility values, as well as domain-specific scores. Ordinary least squares (OLS) regression and Tobit regression were applied to estimate EQ-5D utility values (i.e., UW-mRS), treating mRS scores as dummy variables. Given that utility values are bounded between 0 and 1, we additionally applied alternative approaches including fractional logit, fractional probit, beta regression, and two-part models to model the association between mRS scores and EQ-5D utility values. EQ-5D utility weights were derived from the partial regression coefficients for each mRS category. To assess the influence of covariates on these coefficients, we adjusted for age, sex, hypertension, diabetes mellitus, dyslipidemia, atrial fibrillation, smoking history, alcohol consumption history, BMI, stroke history, pre-stroke functional dependency, ischemic stroke subtype, neurological severity, and time from onset to assessment. Predictive accuracy of each model was evaluated using R-squared (R^2^), root mean square error (RMSE), and mean absolute error (MAE).

To assess how results varied when applying value sets from other countries, we estimated UW-mRS scores by applying several national value sets (China [27], Korea [28], Taiwan [29], the United States [30], and England [31]) to the EQ-5D-5L responses obtained in this study. Additionally, the EQ-5D-5L responses were mapped to the 3L version, and the Japanese 3L value set was applied to conduct a 3L crosswalk analysis [32, 33].

For stratified analyses, subgroup evaluations were conducted according to age (< 75 years and ≥ 75 years), sex, pre-stroke functional status (independent and dependent), and neurological severity (minor [NIHSS score ≤ 4] and non-minor [NIHSS score > 4] stroke). Additional subgroup analyses were performed according to the time from stroke onset to assessment (≤ 14 days and > 14 days) and the type of respondent (self-reported and proxy-reported). Effect modification was examined by including interaction terms of each factor × mRS in the model.

Sensitivity analyses were also conducted. Although none of the covariates had missing values, 220 patients were excluded due to incomplete or missing EQ-5D-5L responses. Missing utility values were imputed using multiple imputation by chained equations based on relevant covariates including age, sex, hypertension, diabetes mellitus, dyslipidemia, atrial fibrillation, smoking history, alcohol consumption history, BMI, stroke history, pre-stroke functional dependency, ischemic stroke subtype, neurological severity, and time from onset to assessment. Furthermore, we repeated the analyses after excluding patients with pre-stroke psychiatric or neurological disorders to minimize potential bias that could have affected utility values before stroke onset.

All statistical analyses were performed using R software (version 4.4.0; https://www.r-project.org/), and two-tailed P values of less than 0.05 were considered statistically significant.

## Results

### Background characteristics

The median age (interquartile range) of the 1452 study participants was 75 (67–83) years, and 596 (41.0%) were female. A total of 241 patients (16.6%) had pre-stroke dependency. Patient characteristics stratified by mRS levels are presented in Table 1. As mRS scores increased, patients tended to be older, more frequently female, and more likely to have atrial fibrillation, a history of stroke, and pre-existing functional dependency, while they were less likely to smoke or consume alcohol. With increasing mRS scores, BMI tended to be lower. The distribution of ischemic stroke subtypes varied by mRS levels, with cardioembolism being more frequent among patients with higher mRS scores.

**Table 1:** Background characteristics according to mRS score

	mRS 0 n = 192	mRS 1 n = 449	mRS 2 n = 206	mRS 3 n = 239	mRS 4 n = 250	mRS 5 n = 116	*P*	P_trend_
Age, y	69 (61–77)	70 (62–78)	73 (66–80)	79 (71–84.5)	82 (74–88)	85.5 (79–90)	< 0.001	< 0.001
Female	68 (35.4)	152 (33.9)	75 (36.4)	109 (45.6)	125 (50.0)	67 (57.8)	< 0.001	< 0.001
Body mass index, kg/m2	24 (22–26)	24 (21–26)	23 (20–25)	22 (20–25)	22 (19–24)	21 (18–23)	< 0.001	< 0.001
*Risk factors*								
Hypertension	159 (82.8)	366 (81.5)	175 (85.0)	207 (86.6)	214 (85.6)	96 (82.8)	0.53	0.23
Diabetes mellitus	57 (29.7)	125 (27.9)	73 (35.6)	63 (26.4)	84 (33.6)	34 (29.3)	0.21	0.52
Dyslipidemia	127 (66.1)	282 (62.8)	127 (61.7)	149 (62.3)	147 (58.8)	61 (52.6)	0.24	0.02
Atrial fibrillation	32 (16.7)	78 (17.4)	32 (15.5)	73 (30.5)	73 (29.2)	66 (56.9)	< 0.001	< 0.001
Smoking	120 (62.5)	270 (60.1)	120 (58.5)	118 (49.4)	110 (44.2)	44 (37.9)	< 0.001	< 0.001
Drinking	74 (38.5)	209 (46.5)	79 (38.7)	62 (25.9)	53 (21.5)	21 (18.1)	< 0.001	< 0.001
Previous stroke	17 (8.9)	71 (15.9)	34 (16.6)	58 (24.4)	80 (32.0)	21 (18.1)	< 0.001	< 0.001
Pre-stroke dependency	1 (0.5)	0 (0.0)	3 (1.5)	67 (28.0)	103 (41.2)	67 (57.8)	< 0.001	< 0.001
*Stroke subtype*								
Cardiembolism	31 (16.1)	65 (14.5)	33 (16.0)	66 (27.6)	68 (27.2)	64 (55.2)	< 0.001	< 0.001
*Non-cardiembolism*								
Large-artery atherosclerosis	26 (13.5)	60 (13.4)	38 (18.4)	46 (19.2)	29 (11.6)	11 (9.5)		
Small-vessel occlusion	71 (37.0)	192 (42.8)	81 (39.3)	64 (26.8)	98 (39.2)	12 (10.3)		
Others	64 (33.3)	132 (29.4)	54 (26.2)	63 (26.4)	55 (22.0)	29 (25.0)		
NIHSS score	0 (0–0)	1 (0–1)	1 (1–2)	2 (1–3)	5 (2–8)	15 (10–19)	< 0.001	< 0.001

mRS, modified rankin scale; P_trend_, P for trend; NIHSS, National Institutes of Health Stroke Scale

Data are presented as median (interquartile range) or n (%)

### Relationship between mRS scores and EQ-5D utility values

**Fig. 1:**
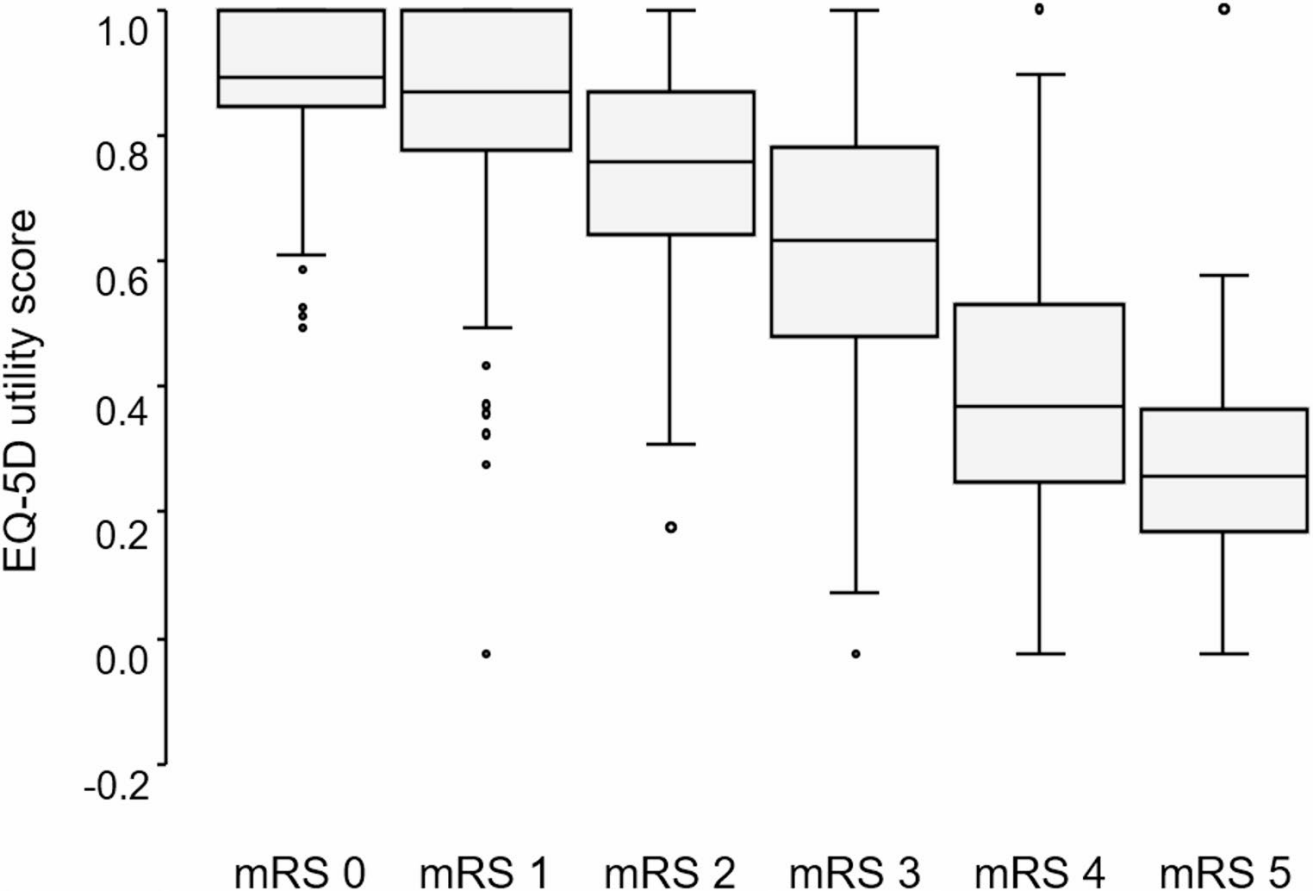
EQ-5D utility values by mRS score. mRS: modified Rankin Scale. This figure shows box plots of EQ-5D utility values by mRS score (0–5). In each box-and-whisker plot, the line within the box represents the median, the box shows the interquartile range, and the whiskers indicate the minimum and maximum values. Outliers are shown as dots and are defined as values greater than 1.5 times the interquartile range beyond the first or third quartile

EQ-5D responses were obtained directly from 1,233 patients (84.9%) and by proxy for 219 patients (15.1%). The mean (SD) EQ-5D utility value for all patients was 0.68 (0.27). The mean (SD) EQ-5D utility values for each mRS score were as follows: mRS 0: 0.90 (0.11), 1: 0.85 (0.14), 2: 0.74 (0.18), 3: 0.62 (0.21), 4: 0.41 (0.21), and 5: 0.28 (0.19). The most substantial decline in utility values was observed between mRS scores 3 and 4 (Fig. 1).

### Relationship between mRS scores and problems in each domain of EQ-5D

**Fig. 2:**
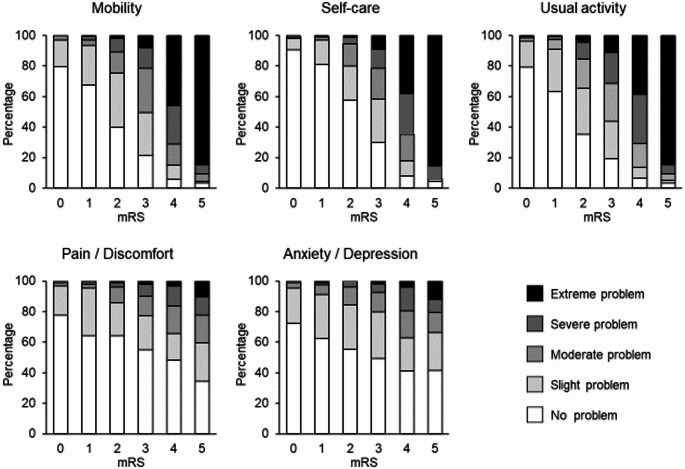
Frequency of problems in five domains by mRS score. mRS: modified Rankin Scale. Distribution of response severity across the five EQ-5D domains by mRS score. Percentages of each severity level are presented for mobility, self-care, usual activities, pain/discomfort, and anxiety/depression

We analyzed the proportion of patients experiencing different levels of problems in each of the five EQ-5D domains according to mRS scores (Fig. 2). Across all domains, the proportion of patients reporting “no problem” decreased, while reports of “severe problem” increased as mRS scores rose from 0 to 5. Severe problems in mobility, self-care, and usual activities were notably more frequent in patients with mRS scores of 4 or higher. In contrast, the increase in problem severity was less marked for pain/discomfort and anxiety/depression. The Spearman’s correlation coefficient between mRS scores and overall EQ-5D utility values was − 0.717, indicating a strong negative correlation. The mRS scores were strongly correlated with self-care, mobility, and usual activities, but showed only weak correlations with pain/discomfort and anxiety/depression (Online Resource 5: Table S1).

### Mapping mRS scores to EQ-5D utility values

We mapped mRS scores to EQ-5D utility values using OLS regression. The estimated utility weights for each mRS level were as follows: mRS 0: 0.90, 1: 0.85, 2: 0.74, 3: 0.62, 4: 0.41, 5: 0.28 (Table 2). Tobit regression model produced similar results, although the estimated utility values were slightly higher for patients with mRS scores of 0–3: mRS 0: 0.97, 1: 0.89, 2: 0.76, 3: 0.63, 4: 0.41, 5: 0.28 (Table 2). To examine whether covariates contributed to the observed changes in regression coefficients and thereby altered the UW-mRS, we individually added variables to the base mRS model and evaluated the resulting changes in partial regression coefficients. Adjustment for neurological severity and age reduced the magnitude of the coefficients. In contrast, adjustment for other factors such as sex, pre-stroke dependency, and time from onset to assessment had minimal impact on the UW-mRS (Table S2). Similarly, the coefficients of mRS scores decreased after adjustment for these covariates in Tobit regression model.

**Table 2:** Association between mRS and EQ-5D utility values

OLS			Tobit		
	B	UW-mRS		B	UW-mRS
Intercept	0.90 (0.88 to 0.92)			0.97 (0.94 to 1.00)	
mRS 0	Reference	0.90		Reference	0.97
mRS 1	− 0.05 (− 0.08 to − 0.02)	0.85		− 0.08 (− 0.12 to − 0.05)	0.89
mRS 2	− 0.16 (− 0.19 to − 0.12)	0.74		− 0.21 (− 0.26 to − 0.17)	0.76
mRS 3	− 0.28 (− 0.31 to − 0.24)	0.62		− 0.34 (− 0.38 to − 0.30)	0.63
mRS 4	− 0.49 (− 0.53 to − 0.46)	0.41		− 0.56 (− 0.60 to − 0.52)	0.41
mRS 5	− 0.62 (− 0.66 to − 0.58)	0.28		− 0.69 (− 0.74 to − 0.64)	0.28
mRS 6	–	0.00		–	0.00

mRS, modified rankin scale score; OLS, ordinary least squares; B, partial regression coefficient; UW-mRS, utility-weighted mRS

OLS regression and Tobit regression were used to estimate the intercept and partial regression coefficients for each mRS score. Point estimates are presented with their 95% confidence intervals. UW-mRS values were derived from the point estimates for each mRS score

We also assessed the model dependency of the UW-mRS. In addition to OLS and Tobit models, we applied fractional logit, fractional probit, beta regression, and two-part models, given that utility values are bounded between 0 and 1. The observed mean values and model-based estimates were closely aligned, with all models yielding values that aligned closely along the diagonal line (Figure S1). In terms of predictive performance, the two-part model showed slightly better accuracy, while the other models—fractional probit, fractional logit, and beta regression—did not differ substantially from the OLS and Tobit models (Table 3).

**Table 3:** Performance of different models in predicting EQ-5D utility values

	OLS	Tobit	Fractional logit	Fractional probit	Beta regression	Two-part model
*Overall cohort*						
R^2^	0.577	0.562	0.577	0.577	0.574	0.691
RMSE	0.174	0.178	0.174	0.174	0.175	0.149
MAE	0.132	0.133	0.132	0.132	0.133	0.099
*Five-fold cross validation*						
R^2^	0.566 ± 0.049	0.550 ± 0.053	0.566 ± 0.049	0.566 ± 0.049	0.561 ± 0.049	0.684 ± 0.025
RMSE	0.175 ± 0.007	0.178 ± 0.006	0.175 ± 0.007	0.175 ± 0.007	0.176 ± 0.006	0.149 ± 0.005
MAE	0.133 ± 0.004	0.134 ± 0.003	0.133 ± 0.004	0.133 ± 0.004	0.134 ± 0.003	0.099 ± 0.003

OLS, ordinary least squares; R^2^, adjusted R-square; RMSE, root mean square error; MAE, mean absolute error

The associations between mRS and utility values were evaluated using OLS, Tobit, fractional logit, fractional probit, beta regression, and two-part models. In the five-fold cross-validation, the mean and standard deviation of R^2^, RMSE, and MAE across the five subsets are presented

We examined the effect of applying value sets from other countries. When tariffs from China [27], Korea [28], Taiwan [29], the United States [30], and England [31] were used, UW-mRS estimates showed considerable variation relative to those derived using the Japanese tariff (Table 4).

**Table 4:** UW-mRS estimated using value sets from different countries

	Japan	China	Korea	Taiwan	United States	England
mRS 0	0.90	0.93	0.90	0.90	0.91	0.93
mRS 1	0.85	0.85	0.85	0.82	0.84	0.85
mRS 2	0.74	0.71	0.76	0.65	0.70	0.75
mRS 3	0.62	0.55	0.65	0.43	0.53	0.62
mRS 4	0.41	0.21	0.41	− 0.04	0.19	0.34
mRS 5	0.28	0.03	0.24	− 0.31	0.00	0.18
mRS 6	0.00	0.00	0.00	0.00	0.00	0.00

UW-mRS, utility-weighted mRS; mRS, modified rankin scale score

UW-mRS values were estimated using OLS regression based on utility values calculated using value sets from multiple countries (China [27], Korea [28], Taiwan [29], United States [30], and England [31]) applied to the results of this study

Additionally, we examined the UW-mRS after converting EQ-5D-5L results to EQ-5D-3L values using the crosswalk method. Re-analysis based on the 3L crosswalk yielded generally lower UW-mRS values across all mRS levels (Table 5).

**Table 5:** Association between mRS and EQ-5D utility values after 3L crosswalk

OLS			Tobit	
	B	UW-mRS	B	UW-mRS
Intercept	0.87 (0.84 to 0.90)		0.94 (0.91 to 0.98)	
mRS 0	Reference	0.87	Reference	0.94
mRS 1	− 0.06 (− 0.09 to − 0.03)	0.81	− 0.09 (− 0.13 to − 0.05)	0.78
mRS 2	− 0.15 (− 0.19 to − 0.12)	0.72	− 0.21 (− 0.26 to − 0.16)	0.66
mRS 3	− 0.25 (− 0.29 to − 0.22)	0.61	− 0.32 (− 0.36 to − 0.28)	0.55
mRS 4	− 0.49 (− 0.53 to − 0.46)	0.38	− 0.57 (− 0.61 to − 0.52)	0.30
mRS 5	− 0.70 (− 0.74 to − 0.66)	0.17	− 0.77 (− 0.83 to − 0.72)	0.10
mRS 6	–	0.00	–	0.00
R^2^	0.564		0.550	
RMSE	0.189		0.192	
MAE	0.147		0.150	

mRS, modified Rankin Scale score; OLS, ordinary least squares; B, partial regression coefficient; UW-mRS, utility-weighted mRS; R^2^, adjusted R-square; RMSE, root mean square error; MAE, mean absolute error

OLS regression and Tobit regression were used to estimate the intercept and partial regression coefficients for each mRS score. Point estimates are presented with their 95% confidence intervals. UW-mRS values were derived from the point estimates for each mRS score. Utility values were calculated by first mapping the 5L responses onto the 3L system and then applying the Japanese 3L tariff [32, 33]

### Stratified analyses

We next examined whether the associations between mRS and utility values differed according to age (< 75 years and ≥ 75 years, Table S3), sex (Table S4), pre-stroke functional status (independent and dependent, Table S5), and neurological severity (NIHSS score ≤ 4 vs. > 4, Table S6). Interaction terms were statistically significant for pre-stroke functional status in the OLS model and for age in the Tobit model. UW-mRS values were relatively low for mRS scores 0–1 among patients aged ≥ 75 years and tended to be lower across all mRS levels among patients with pre-stroke dependency.

Because the evaluations in this study were conducted shortly after acute treatment, the interval between stroke onset and assessment was relatively short, although it varied among patients. When stratified by assessment timing (≤ 14 vs. > 14 days), the interaction term between mRS and timing was significant, with slightly higher UW-mRS values observed in the > 14-day group than in the ≤ 14-day group (Table S7).

Mean (SD) utility values for patients whose EQ-5D-5L responses were completed by proxies (mRS 0: 0.75 [0.23], 1: 0.68 [0.11], 2: 0.52 [0.20], 3: 0.54 [0.17], 4: 0.33 [0.15], 5: 0.24 [0.13]) were lower than those for patient-completed responses, particularly for mRS scores 3–5 (mRS 0: 0.90 [0.11], 1: 0.85 [0.14], 2: 0.75 [0.17], 3: 0.64 [0.21], 4: 0.44 [0.23], 5: 0.42 [0.29]). However, interaction terms between mRS and respondent type were not statistically significant in either model (Table S8).

### Sensitivity analysis

A total of 220 patients were excluded due to missing or incomplete EQ-5D-5L responses; no missing values were observed for any covariates. These excluded patients had higher mRS scores than those included (Table S9). After multiple imputation of missing utility values, the UW-mRS estimates were largely unchanged (Table S10). To further address potential bias due to psychiatric or neurological disorders, we repeated the analyses after excluding patients with pre-stroke dementia, Parkinsonism, or depression (Table S11). The UW-mRS estimates were not materially affected by these exclusions.

## Discussion

### Patient characteristics and utility values

We observed a strong inverse relationship between mRS scores and EQ-5D utility values, consistent with previous reports [15]. Compared with clinical trial cohorts, our study population was older and included a higher proportion of patients with pre-stroke dependency (16.6% vs. 7.4% in pooled trial data [21]). As pre-stroke dependency is independently associated with lower EQ-5D values [35], our results may more accurately reflect real-world stroke populations. With global population aging, particular attention should be paid to older patients, who often present with multimorbidity and pre-stroke dependency [2].

Previous studies have shown that several factors, such as age [35–41], sex [42–44], BMI [43], neurological severity [35–37, 40–42, 44–46], cardiovascular risk factors [39, 41, 43, 47], and pre-stroke ADL dependency [35, 40] are associated with lower QOL after stroke. In our analysis, adjustment for age and neurological severity—both key determinants of post-stroke QOL—resulted in reduced regression coefficients for mRS scores, especially for mRS 3–5. These findings suggest that age and neurological severity may partly mediate the association between mRS and utility values.

We also examined the correlations between individual EQ-5D domains and mRS. Mobility, self-care, and usual activities showed strong correlations, whereas pain/discomfort and anxiety/depression demonstrated weaker associations. This pattern aligns with prior studies showing that stroke predominantly affects physical domains and that the mRS effectively captures these impairments [35]. In contrast, non-physical domains are influenced by broader psychosocial factors, which may account for the variability in utilities within each mRS category. Notably, some patients with severe disability reported “no problems” on EQ-5D-5L. While the mRS was evaluated by trained raters with adjudicated validity, the EQ-5D reflected patient self-reports. Therefore, the health states reported by patients may have differed from their objectively assessed clinical condition. Further investigation is warranted to elucidate the underlying reasons for this discrepancy, including the potential influence of cognitive function and neurological symptoms.

### Factors influencing UW-mRS

The UW-mRS estimates from OLS were as follows: mRS 0: 0.90, 1: 0.85, 2: 0.74, 3: 0.62, 4: 0.41, and 5: 0.28. Comparison with previous reports and meta-analysis indicated that values for mRS 0–3 were similar, whereas values for mRS 4–5 were higher in our study (Figure S2). When value sets from other countries were applied, substantial variability in UW-mRS estimates was observed, particularly among patients with severe disability. This finding underscores how utility values depend on the choice of tariff and reflect cultural and systemic differences in health perceptions [18, 20, 22]. For example, Asian patients have been reported to describe better health-related QOL after stroke, partly due to strong family support systems [18, 34]. However, regional variations were evident even within Asia. Values based on the Korean tariff were similar to ours, whereas those derived from the Taiwanese tariff were lower than those obtained using Western tariffs. Conversely, UW-mRS values based on the Japanese tariff were consistently higher than those derived from the United States and England. This may reflect Japan’s universal health coverage and long-term care insurance system, which could foster more positive perceptions of health even among individuals with severe disability [48, 49].

We also compared different mapping methods. When the 3L crosswalk was applied, UW-mRS values were consistently lower than those estimated using the 5L version, highlighting the impact of instrument selection. We additionally applied alternative modeling approaches, including fractional logit, fractional probit, beta regression, and two-part models. Predictive performance was broadly comparable across these approaches, and the predicted UW-mRS values were generally consistent, indicating that model choice exerted only a minor influence compared with other sources of variation.

Stratified analyses suggested the presence of effect modification. The interaction between age and mRS was significant in the Tobit regression, while the interaction between pre-stroke functional status and mRS was significant in the OLS regression, suggesting that these factors modify the relationship between mRS and utility values. UW-mRS estimates also varied according to the time from stroke onset to assessment, with higher values observed at later assessments. Although previous studies have reported that UW-mRS remains stable over time [16], our findings imply that this measure may shift depending on the timing of assessment.

Utility values derived from self-reported health status were higher for mRS scores 3–5 than those based on proxy reports. Proxies—often primary caregivers—tend to rate patients’ health states lower than patients do themselves, a discrepancy also documented in prior studies [34, 37]. However, the interaction between mRS and respondent type was not statistically significant in our study. Given that the mRS has only six grades (0–5), whereas the EQ-5D-5L provides more than 100 distinct utility scores, some differences between patient- and proxy-reported responses may reflect true variation in health status rather than reporting bias. Further research is warranted to clarify the impact of respondent type on UW-mRS after stroke.

### Future directions

EQ-5D-derived utilities are essential for cost-effectiveness analyses, yet few real-world stroke studies have directly measured EQ-5D-5L utilities. Because most existing datasets do not include utilities or quality-adjusted life years (QALYs), economic evaluations have therefore been limited. In contrast, the mRS is almost universally collected in stroke research. Mapping mRS scores to utility values thus provides a practical alternative for estimating QALYs when direct measurements are unavailable.

The largest decline in utility values occurred between mRS scores 3 and 4, consistent with findings from previous trial cohorts [21, 34]. This suggests that patients with severe disability may have the greatest potential to achieve QOL gains, underscoring the importance of therapeutic interventions in this group. Including older patients and those with pre-stroke dependency in clinical trials is also critical, as these individuals may derive particular benefit from targeted interventions.

Our results demonstrated that UW-mRS values varied according to age, pre-stroke functional status, timing of assessment, and the choice of tariff. Furthermore, mapping via the 3L crosswalk yielded lower estimates than the 5L version, highlighting the importance of accounting for differences between EQ-5D versions. Collectively, these findings indicate that the UW-mRS is not easily generalizable and should preferably be estimated in a population- and country-specific manner. Although the UW-mRS remains a useful and practical approach, it should be considered a secondary option. Direct measurement of EQ-5D utilities should be incorporated in future stroke research to enable more robust and generalizable economic evaluations.

## Limitations

This study has several limitations. First, although we aimed for comprehensive inclusion, some patients either did not respond or provided incomplete answers, which may have led to missing utility values and potential selection bias. However, our multiple imputation analysis indicated no material effect on the estimates. Second, both the mRS and QOL were assessed during hospitalization, and results may have varied depending on the timing of assessment. As post-stroke QOL tends to improve over time [22, 50, 51], the applicability of UW-mRS in the acute phase is limited, and reassessment in the chronic phase is warranted. Third, approximately 15% of EQ-5D responses were proxy-reported. Although such use is common in stroke research, discrepancies between patient- and proxy-reported assessments have been documented [11, 52, 53]. Fourth, pre-stroke conditions such as dementia, Parkinsonism, or depression could have influenced results, although sensitivity analyses excluding these patients showed no meaningful changes in UW-mRS estimates. Finally, as the FSR is confined to a single region of Japan, the generalizability of our findings may be restricted. Validation in more recent Japanese cohorts and external populations is needed.

## Conclusions

This study of Japanese patients with ischemic stroke demonstrated the relationship between functional status and utility values and provided mRS-based estimates for economic evaluations. While the mRS remains the standard outcome measure in stroke research, the UW-mRS provides a utility-based assessment framework and represents a practical, though secondary, approach when direct EQ-5D measurement is unavailable.

## Supplementary Information

Below is the link to the electronic supplementary material.

Supplementary file1 (PDF 681 kb)

## Declarations

### Data availability

Because of the sensitive nature of the data, the datasets supporting the findings of this study are available from the corresponding author upon reasonable request by qualified researchers trained in human subject confidentiality protocols.
