# Supplementary material for: Utility-weighted modified rankin scale scores in patients with ischemic stroke: a multicenter observational study
Source: Qual Life Res. 2026 Jan 9;35(2):26. doi: 10.1007/s11136-025-04114-7 (PMC12789241; doi:10.1007/s11136-025-04114-7)
Supplement: Supplementary file 1 — Supplementary file1 (PDF 681 kb) [file 11136_2025_4114_MOESM1_ESM.pdf]

Quality of Life Research

Supplementary Material

Utility-weighted modified Rankin Scale scores in patients with ischemic stroke: A multicenter observational study

Fumi Irie, MD, PhD[a,b,c]; Koutarou Matsumoto, MPH, PhD[a]; Ryu Matsuo, MD, PhD[a,b,c]; Yoshinobu Wakisaka, MD, PhD[b,c]; Tetsuro Ago, MD, PhD[b,c]; Takanari Kitazono, MD, PhD[b,c]; Masahiro Kamouchi, MD, PhD[a,c]; on behalf of the Fukuoka Stroke Registry Investigators

[a]Department of Health Care Administration and Management, Graduate School of Medical Sciences, Kyushu University, Fukuoka, Japan
[b]Department of Medicine and Clinical Science, Graduate School of Medical Sciences, Kyushu University, Fukuoka, Japan
[c]Center for Cohort Studies, Graduate School of Medical Sciences, Kyushu University, Fukuoka, Japan

Corresponding author: Masahiro Kamouchi, MD, PhD
Department of Health Care Administration and Management, Graduate School of Medical Sciences, Kyushu University, 3-1-1 Maidashi, Higashi-ku, Fukuoka 812-8582, Japan
E-mail: kamouchi.masahiro.736@m.kyushu-u.ac.jp

Supplemental Methods
Supplemental Figures
Figure S1. Relationship between observed and predicted UW-mRS values across different models
Figure S2. Relationship between the UW-mRS estimates from this study and those reported in previous studies
Supplemental Tables
Table S1. Correlations between mRS and EQ-5D utility values or individual domain scores
Table S2. Association between mRS and EQ-5D utility values after adjustment for individual covariates
Table S3. Association between mRS and EQ-5D utility values stratified by age
Table S4. Association between mRS and EQ-5D utility values stratified by sex
Table S5 Association between mRS and EQ-5D utility values stratified by pre-stroke functional status
Table S6. Association between mRS and EQ-5D utility values stratified by neurological severity
Table S7. Association between mRS and EQ-5D utility values stratified by time from onset to assessment
Table S8. Association between mRS and EQ-5D utility values stratified by respondents
Table S9. Background characteristics of included and excluded patients
Table S10. Association between mRS and EQ-5D utility values before and after multiple imputations
Table S11. Association between mRS and EQ-5D utility values after excluding patients with pre-stroke dementia, Parkinsonism, or depression
Appendix

Supplemental Methods

Hypertension was defined as systolic blood pressure ≥140 mmHg or diastolic pressure ≥90 mmHg in the chronic stage, or as current use of antihypertensive medication. Diabetes mellitus was determined according to the diagnostic criteria of the Japan Diabetes Society [1] in the chronic stage or by a documented medical history of diabetes mellitus. Dyslipidemia was defined as any of the following: low-density lipoprotein cholesterol ≥3.62 mmol/L, high-density lipoprotein cholesterol <1.03 mmol/L, triglycerides ≥1.69 mmol/L, or current use of a cholesterol-lowering drug. Atrial fibrillation was diagnosed based on electrocardiographic findings at admission or during hospitalization. Smoking and drinking history included both current and past habits prior to the index stroke. Body mass index was measured at admission. Stroke history included both ischemic and hemorrhagic events prior to the index stroke. Pre-stroke functional dependency was determined at admission based on interviews with the patient or their family, mapped to the modified Rankin Scale, and defined as dependent for scores of 2 or higher. Ischemic stroke was classified into four subtypes according to the TOAST (Trial of ORG 10172 in Acute Stroke Treatment) criteria [2]. Neurological severity was assessed using the National Institutes of Health Stroke Scale, which ranges from 0 (no neurological deficits) to 42 (most severe deficits) [3].

References

1. Shinohara, Y., Minematsu, K., Amano, T., Ohashi, Y., & mRS Reliability Study Group. (2007). Reliability of modified Rankin Scale -Introduction of a guidance scheme and a questionnaire written in Japanese-. Jpn J Stroke, 29(1), 6-13.
2. Adams, H. P., Jr., Bendixen, B. H., Kappelle, L. J., Biller, J., Love, B. B., Gordon, D. L., & Marsh, E. E., 3rd. (1993). Classification of subtype of acute ischemic stroke. Definitions for use in a multicenter clinical trial. TOAST. Trial of Org 10172 in Acute Stroke Treatment. Stroke, 24(1), 35-41.
3. Brott, T., Adams, H. P., Jr., Olinger, C. P., Marler, J. R., Barsan, W. G., Biller, J., Spilker, J., Holleran, R., Eberle, R., Hertzberg, V., Rorick, M., Moomaw, C. J., & Walker, M. (1989). Measurements of acute cerebral infarction: a clinical examination scale. Stroke, 20(7), 864-870.

Figure S1. Relationship between observed and predicted UW-mRS values across different models

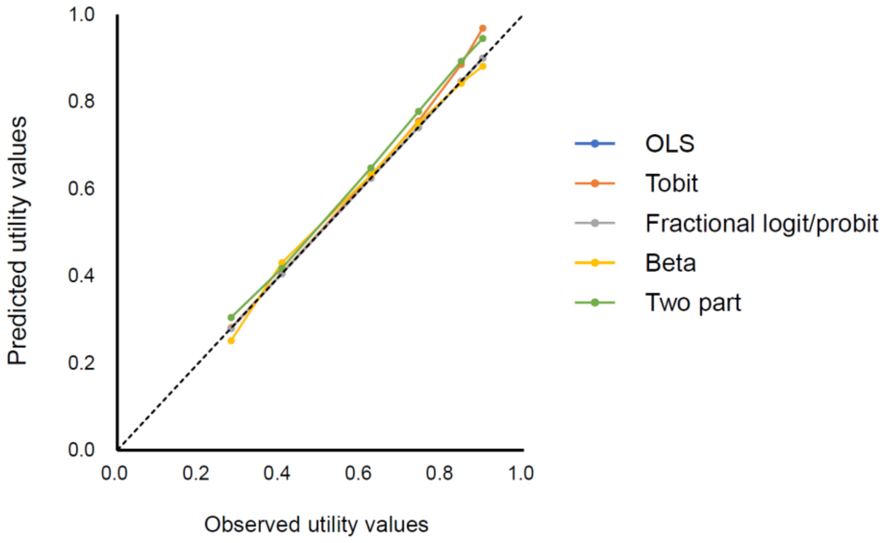

UW-mRS: utility-weighted mRS, OLS: ordinary least squares.

The figure shows the relationship between the observed values in each mRS patient group and the UW-mRS estimated using OLS, Tobit, fractional logit/probit, beta, and two-part models. For each mRS category, observed mean values are shown on the x-axis and model-based UW-mRS estimates on the y-axis. Observed values represent the mean utility values for patients within each mRS group.

Figure S2. Relationship between the UW-mRS estimates from this study and those reported in previous studies

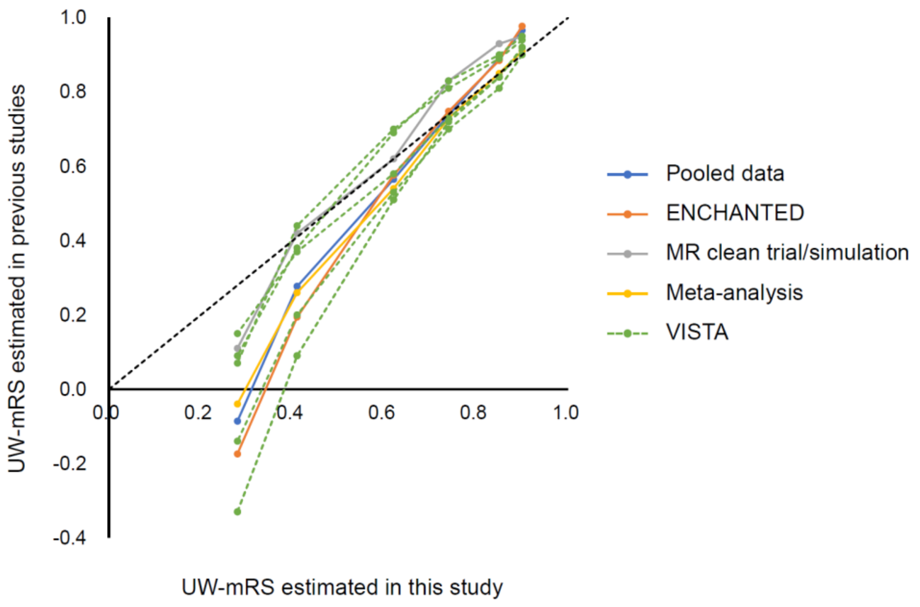

UW-mRS: utility-weighted mRS.
UW-mRS values estimated in this study were compared with those reported in previous studies of ischemic stroke. The horizontal axis shows UW-mRS values estimated in this study, while the vertical axis shows UW-mRS values reported in previous studies.
Pooled data: Combined datasets from INTERACT, ENCHANTED, HeadPoST, ATTEND, SCAST, and COSSACS, with outcomes assessed at 3–6 months (mRS assessed at 2 weeks in COSSACS) [1]. ENCHANTED: Data from the ENCHANTED trial (including both Asian and non-Asian countries), assessed at 3 months [2]. MR CLEAN trial/simulation: Data from the MR CLEAN trial/simulation (Netherlands), assessed at 3 months [3]. Meta-analysis: Datasets from previous studies, with outcomes assessed from within 1 month to more than 24 months [4]. VISTA: Data from the VISTA database (36 participating countries), assessed at 3 months. Utility values were estimated using value sets from China, Germany, Poland, Spain, England, and the United States [5].

References

1. Wang, X., Moullaali, T. J., Li, Q., Berge, E., Robinson, T. G., Lindley, R., Zheng, D., Delcourt, C., Arima, H., Song, L., Chen, X., Yang, J., Chalmers, J., Anderson, C. S., & Sandset, E. C. (2020). Utility-weighted modified Rankin scale scores for the assessment of stroke outcome: Pooled analysis of 20,000+ patients. Stroke, 51(8), 2411-2417.
2. Chen, X., Wang, X., Delcourt, C., Li, J., Arima, H., Hackett, M. L., Robinson, T., Lavados, P. M., Lindley, R. I., Chalmers, J., Anderson, C. S., & ENCHANTED Investigators. (2020). Ethnicity and other determinants of quality of functional outcome in acute ischemic stroke: The ENCHANTED Trial. Stroke, 51(2), 588-593.
3. Dijkland, S. A., Voormolen, D. C., Venema, E., Roozenbeek, B., Polinder, S., Haagsma, J. A., Nieboer, D., Chalos, V., Yoo, A. J., Schreuders, J., van der Lugt, A., Majoie, C., Roos, Y., van Zwam, W. H., van Oostenbrugge, R. J., Steyerberg, E. W., Dippel, D. W. J., Lingsma, H. F., & MR CLEAN Investigators. (2018). Utility-weighted modified Rankin scale as primary outcome in stroke trials: A simulation study. Stroke, 49(4), 965-971.
4. Zhou, J., Wei, Q., Hu, H., Liu, W., Guan, X., Ma, A., & Wang, L. (2023). A systematic review and meta-analysis of health utility values among patients with ischemic stroke. Front Neurol, 14, 1219679.
5. Ali, M., MacIsaac, R., Quinn, T. J., Bath, P. M., Veenstra, D. L., Xu, Y., Brady, M. C., Patel, A., & Lees, K. R. (2017). Dependency and health utilities in stroke: Data to inform cost-effectiveness analyses. Eur Stroke J, 2(1), 70-76.

Table S1. Correlations between mRS and EQ-5D utility values or individual domain scores

	ρ
EQ-5D utility values	-0.717
Scores in each domain of EQ-5D	
Self-care	0.725
Mobility	0.715
Usual activity	0.707
Pain/discomfort	0.281
Anxiety/depression	0.266

mRS: modified Rankin Scale score, ρ: Spearman rank correlation coefficient.
Spearman's rank correlation coefficients (ρ) were calculated between mRS scores and EQ-5D utility values, as well as between mRS and each EQ-5D domain score. The mRS scores increase with greater disability, and each EQ-5D domain was rated on a five-level scale ranging from 0 ("no problem") to 4 ("extreme problem").

Table S2. Association between mRS and EQ-5D utility values after adjustment for individual covariates

	Stroke severity		Age		Sex		Pre-stroke dependency		Time from onset to assessment	
	B	UW-mRS	B	UW-mRS	B	UW-mRS	B	UW-mRS	B	UW-mRS
OLS										
Intercept	0.90 (0.88 to 0.92)		0.92 (0.86 to 0.98)		0.90 (0.88 to 0.93)		0.90 (0.88 to 0.92)		0.91 (0.88 to 0.93)	
mRS 0	Reference	0.90	Reference	0.92	Reference	0.90	Reference	0.90	Reference	0.91
mRS 1	-0.05 (-0.08 to -0.02)	0.85	-0.05 (-0.08 to -0.02)	0.87	-0.05 (-0.08 to -0.02)	0.85	-0.05 (-0.08 to -0.02)	0.85	-0.05 (-0.08 to -0.02)	0.85
mRS 2	-0.15 (-0.18 to -0.11)	0.75	-0.16 (-0.19 to -0.12)	0.77	-0.16 (-0.19 to -0.12)	0.75	-0.16 (-0.19 to -0.12)	0.74	-0.16 (-0.19 to -0.12)	0.75
mRS 3	-0.26 (-0.29 to -0.23)	0.64	-0.27 (-0.31 to -0.24)	0.65	-0.27 (-0.31 to -0.24)	0.63	-0.27 (-0.31 to -0.24)	0.63	-0.27 (-0.31 to -0.24)	0.64
mRS 4	-0.46 (-0.50 to -0.42)	0.44	-0.49 (-0.52 to -0.46)	0.43	-0.49 (-0.53 to -0.46)	0.41	-0.49 (-0.53 to -0.45)	0.41	-0.49 (-0.52 to -0.46)	0.42
mRS 5	-0.52 (-0.58 to -0.46)	0.38	-0.61 (-0.66 to -0.57)	0.31	-0.62 (-0.66 to -0.58)	0.29	-0.61 (-0.66 to -0.57)	0.29	-0.61 (-0.65 to -0.57)	0.29
mRS 6	–	0.00	–	0.00	–	0.00	–	0.00	–	0.00
Covariate	-0.03 (-0.05 to -0.02)		0.00 (-0.01 to 0.00)		-0.01 (-0.03 to 0.01)		-0.01 (-0.03 to 0.01)		-0.02 (-0.05 to 0.01)	
R^2	0.582		0.577		0.578		0.577		0.577	
RMSE	0.173		0.174		0.174		0.174		0.175	
MAE	0.132		0.132		0.132		0.132		0.132	
Tobit										
Intercept	0.97 (0.94 to 1.00)		1.00 (0.93 to 1.07)		0.97 (0.94 to 1.01)		0.97 (0.94 to 1.00)		0.98 (0.94 to 1.01)	
mRS 0	Reference	0.97	Reference	1.00	Reference	0.97	Reference	0.97	Reference	0.98
mRS 1	-0.08 (-0.11 to -0.04)	0.89	-0.08 (-0.12 to -0.05)	0.92	-0.08 (-0.12 to -0.05)	0.89	-0.08 (-0.12 to -0.04)	0.89	-0.08 (-0.12 to -0.05)	0.90
mRS 2	-0.20 (-0.24 to -0.16)	0.77	-0.21 (-0.25 to -0.17)	0.79	-0.21 (-0.25 to -0.17)	0.76	-0.21 (-0.25 to -0.17)	0.76	-0.21 (-0.25 to -0.17)	0.77

mRS 3	-0.32 (-0.36 to -0.28)	0.65	-0.33 (-0.38 to -0.29)	0.67	-0.34 (-0.38 to -0.30)	0.64	-0.33 (-0.38 to -0.29)	0.63	-0.33 (-0.38 to -0.29)	0.64
mRS 4	-0.53 (-0.57 to -0.48)	0.44	-0.56 (-0.60 to -0.51)	0.44	-0.56 (-0.60 to -0.52)	0.42	-0.56 (-0.60 to -0.51)	0.41	-0.56 (-0.60 to -0.51)	0.42
mRS 5	-0.58 (-0.65 to -0.51)	0.39	-0.68 (-0.73 to -0.63)	0.32	-0.68 (-0.73 to -0.63)	0.29	-0.68 (-0.73 to -0.63)	0.29	-0.68 (-0.73 to -0.62)	0.30
mRS 6	–	0.00	–	0.00	–	0.00	–	0.00	–	0.00
Covariate	-0.04 (-0.05 to -0.02)		0.00 (-0.01 to 0.00)		-0.02 (-0.04 to 0.00)		-0.01 (-0.03 to 0.02)		-0.02 (-0.06 to 0.01)	
R^2	0.569		0.564		0.564		0.564		0.562	
RMSE	0.176		0.178		0.177		0.178		0.178	
MAE	0.133		0.133		0.133		0.133		0.134	

mRS: modified Rankin Scale score, B: partial regression coefficient, UW-mRS: utility-weighted mRS, OLS: ordinary least squares, R^2: adjusted R-square, RMSE: root mean square error, MAE: mean absolute error.

OLS regression and Tobit regression were used to estimate the intercept and partial regression coefficients for each variable. Point estimates were calculated for each mRS score and for each covariate (stroke severity per 5 National Institutes of Health Stroke Scale points, age per 10 years, sex, pre-stroke dependency, and time from onset to assessment per 30 days) and are presented with their 95% confidence intervals. UW-mRS values were derived from the point estimates for each mRS score after adjusting for each covariate.

Table S3. Association between mRS and EQ-5D utility values stratified by age

	<75 y		≥75 y		
	B	UW-mRS	B	UW-mRS	$P_{interaction}$
OLS					
Intercept	0.91 (0.89 to 0.94)		0.87 (0.83 to 0.92)		
mRS 0	Reference	0.91	Reference	0.87	0.21
mRS 1	-0.04 (-0.08 to -0.01)	0.87	-0.06 (-0.12 to -0.01)	0.81	
mRS 2	-0.17 (-0.21 to -0.13)	0.74	-0.13 (-0.19 to -0.07)	0.74	
mRS 3	-0.29 (-0.33 to -0.24)	0.63	-0.25 (-0.30 to -0.20)	0.62	
mRS 4	-0.56 (-0.60 to -0.51)	0.36	-0.45 (-0.50 to -0.40)	0.42	
mRS 5	-0.65 (-0.73 to -0.56)	0.27	-0.59 (-0.65 to -0.53)	0.28	
mRS 6	–	0.00	–	0.00	
R^2	0.564		0.531		
RMSE	0.158		0.185		
MAE	0.119		0.142		
Tobit					
Intercept	0.99 (0.95 to 1.03)		0.92 (0.87 to 0.98)		
mRS 0	Reference	0.99	Reference	0.92	0.01
mRS 1	-0.07 (-0.12 to -0.03)	0.91	-0.09 (-0.15 to -0.02)	0.84	
mRS 2	-0.24 (-0.29 to -0.18)	0.75	-0.17 (-0.23 to -0.10)	0.76	
mRS 3	-0.36 (-0.42 to -0.30)	0.63	-0.30 (-0.36 to -0.23)	0.63	
mRS 4	-0.63 (-0.69 to -0.57)	0.36	-0.50 (-0.56 to -0.44)	0.42	
mRS 5	-0.72 (-0.82 to -0.61)	0.27	-0.64 (-0.71 to -0.57)	0.28	
mRS 6	–	0.00	–	0.00	
R^2	0.530		0.525		
RMSE	0.164		0.186		
MAE	0.122		0.143		

mRS: modified Rankin Scale score, B: partial regression coefficient, UW-mRS: utility-weighted mRS, $P_{interaction}$: P for interaction, OLS: ordinary least squares, R^2: adjusted R-square, RMSE: root mean square error, MAE: mean absolute error.

Patients were stratified into two groups according to age (≤75 years and ≥75 years). OLS regression and Tobit regression were used to estimate the intercept and partial regression coefficients for each mRS score. Point estimates are presented with their 95% confidence intervals. UW-mRS values were derived from the point estimates for each mRS score. The P value for interaction was evaluated by adding an interaction term of mRS × age group in the model.

Table S4. Association between mRS and EQ-5D utility values stratified by sex

	Female		Male		
	B	UW-mRS	B	UW-mRS	$P_{interaction}$
OLS					
Intercept	0.90 (0.86 to 0.95)		0.90 (0.87 to 0.93)		
mRS 0	Reference	0.90	Reference	0.90	0.79
mRS 1	-0.08 (-0.13 to -0.03)	0.83	-0.04 (-0.08 to 0.00)	0.86	
mRS 2	-0.17 (-0.22 to -0.11)	0.74	-0.15 (-0.20 to -0.11)	0.74	
mRS 3	-0.30 (-0.36 to -0.25)	0.60	-0.25 (-0.30 to -0.21)	0.64	
mRS 4	-0.49 (-0.54 to -0.44)	0.42	-0.50 (-0.55 to -0.46)	0.39	
mRS 5	-0.63 (-0.69 to -0.57)	0.27	-0.61 (-0.67 to -0.55)	0.29	
mRS 6	–	0.00	–	0.00	
R^2	0.589		0.555		
RMSE	0.174		0.174		
MAE	0.134		0.131		
Tobit					
Intercept	0.97 (0.92 to 1.02)		0.97 (0.93 to 1.01)		
mRS 0	Reference	0.97	Reference	0.97	0.72
mRS 1	-0.12 (-0.17 to -0.06)	0.85	-0.07 (-0.11 to -0.02)	0.90	
mRS 2	-0.22 (-0.28 to -0.15)	0.75	-0.21 (-0.27 to -0.16)	0.76	
mRS 3	-0.36 (-0.43 to -0.30)	0.60	-0.32 (-0.37 to -0.26)	0.65	
mRS 4	-0.55 (-0.61 to -0.49)	0.42	-0.57 (-0.63 to -0.52)	0.39	
mRS 5	-0.69 (-0.76 to -0.63)	0.27	-0.68 (-0.75 to -0.60)	0.29	
mRS 6	–	0.00	–	0.00	
R^2	0.580		0.532		
RMSE	0.176		0.178		
MAE	0.135		0.133		

mRS: modified Rankin Scale score, B: partial regression coefficient, UW-mRS: utility-weighted mRS, $P_{interaction}$: P for interaction, OLS: ordinary least squares, R^2: adjusted R-square, RMSE: root mean square error, MAE: mean absolute error.

Patients were stratified into two groups according to sex. OLS regression and Tobit regression were used to estimate the intercept and partial regression coefficients for each mRS score. Point estimates are presented with their 95% confidence intervals. UW-mRS values were derived from the point estimates for each mRS score. The P value for interaction was evaluated by adding an interaction term of mRS × sex in the model.

Table S5 Association between mRS and EQ-5D utility values stratified by pre-stroke functional status

	Pre-stroke independent		Pre-stroke dependent		
	B	UW-mRS	B	UW-mRS	$P_{interaction}$
OLS					
Intercept	0.90 (0.88 to 0.92)		0.82 (0.61 to 1.04)		
mRS 0	Reference	0.90	Reference	0.82	<0.001
mRS 1	-0.05 (-0.08 to -0.02)	0.85	0.00 (-0.23 to 0.22)	0.82	
mRS 2	-0.15 (-0.19 to -0.11)	0.75	-0.10 (-0.32 to 0.12)	0.72	
mRS 3	-0.30 (-0.33 to -0.26)	0.60	-0.18 (-0.40 to 0.04)	0.64	
mRS 4	-0.50 (-0.54 to -0.46)	0.40	-0.42 (-0.64 to -0.20)	0.41	
mRS 5	-0.57 (-0.64 to -0.51)	0.33	-0.56 (-0.78 to -0.34)	0.26	
mRS 6	–	0.00	–	0.00	
R^2	0.506		0.495		
RMSE	0.164		0.191		
MAE	0.123		0.149		
Tobit					
Intercept	0.97 (0.94 to 1.00)		0.82 (0.59 to 1.06)		
mRS 0	Reference	0.97	Reference	0.82	0.08
mRS 1	-0.08 (-0.12 to -0.04)	0.89	0.04 (-0.20 to 0.28)	0.86	
mRS 2	-0.21 (-0.25 to -0.16)	0.76	-0.09 (-0.33 to 0.15)	0.74	
mRS 3	-0.36 (-0.41 to -0.31)	0.61	-0.17 (-0.41 to 0.06)	0.65	
mRS 4	-0.56 (-0.62 to -0.51)	0.41	-0.42 (-0.65 to -0.18)	0.41	
mRS 5	-0.64 (-0.72 to -0.56)	0.33	-0.56 (-0.80 to -0.32)	0.27	
mRS 6	–	0.00	–	0.00	
R^2	0.477		0.492		
RMSE	0.169		0.191		
MAE	0.125		0.149		

mRS: modified Rankin Scale score, B: partial regression coefficient, UW-mRS: utility-weighted mRS, $P_{interaction}$: P for interaction, OLS: ordinary least squares, R^2: adjusted R-square, RMSE: root mean square error, MAE: mean absolute error.

Patients were stratified into two groups according to pre-stroke functional status (independent and dependent). OLS regression and Tobit regression were used to estimate the intercept and partial regression coefficients for each mRS score. Point estimates are presented with their 95% confidence intervals. UW-mRS values were derived from the point estimates for each mRS score. The P value for interaction was evaluated by adding an interaction term of mRS × pre-stroke functional status in the model.

Table S6. Association between mRS and EQ-5D utility values stratified by neurological severity

	Minor stroke		Non-minor stroke		
	B	UW-mRS	B	UW-mRS	$P_{interaction}$
OLS					
Intercept	0.90 (0.88 to 0.93)		0.49 (0.12 to 0.87)		
mRS 0	Reference	0.90	Reference	0.49	0.56
mRS 1	-0.05 (-0.08 to -0.03)	0.85	0.25 (-0.28 to 0.77)	0.74	
mRS 2	-0.16 (-0.19 to -0.13)	0.74	0.07 (-0.38 to 0.53)	0.57	
mRS 3	-0.26 (-0.29 to -0.22)	0.65	0.01 (-0.36 to 0.39)	0.51	
mRS 4	-0.46 (-0.49 to -0.42)	0.45	-0.13 (-0.50 to 0.24)	0.37	
mRS 5	-0.57 (-0.67 to -0.47)	0.33	-0.22 (-0.59 to 0.15)	0.27	
mRS 6	–	0.00	–	0.00	
R^2	0.416		0.143		
RMSE	0.168		0.186		
MAE	0.128		0.138		
Tobit					
Intercept	0.97 (0.94 to 1.00)		0.49 (0.12 to 0.87)		
mRS 0	Reference	0.97	Reference	0.49	0.12
mRS 1	-0.09 (-0.12 to -0.05)	0.89	0.25 (-0.28 to 0.77)	0.74	
mRS 2	-0.21 (-0.26 to -0.17)	0.76	0.07 (-0.38 to 0.53)	0.57	
mRS 3	-0.32 (-0.36 to -0.28)	0.65	0.01 (-0.36 to 0.39)	0.51	
mRS 4	-0.52 (-0.57 to -0.48)	0.45	-0.13 (-0.50 to 0.25)	0.37	
mRS 5	-0.64 (-0.76 to -0.51)	0.34	-0.22 (-0.59 to 0.16)	0.28	
mRS 6	–	0.00	–	0.00	
R^2	0.386		0.143		
RMSE	0.173		0.186		
MAE	0.130		0.138		

mRS: modified Rankin Scale score, B: partial regression coefficient, UW-mRS: utility-weighted mRS, $P_{interaction}$: P for interaction, OLS: ordinary least squares, R^2: adjusted R-square, RMSE: root mean square error, MAE: mean absolute error.

Patients were stratified into two groups according to neurological severity (minor and non-minor stroke). OLS regression and Tobit regression were used to estimate the intercept and partial regression coefficients for each mRS score. Point estimates are presented with their 95% confidence intervals. UW-mRS values were derived from the point estimates for each mRS score. The P value for interaction was evaluated by adding an interaction term of mRS × neurological severity in the model.

Table S7. Association between mRS and EQ-5D utility values stratified by time from onset to assessment

	≤14 days		>14 days		
	B	UW-mRS	B	UW-mRS	$P_{interaction}$
OLS					
Intercept	0.90 (0.87 to 0.92)		0.91 (0.86 to 0.96)		
mRS 0	Reference	0.90	Reference	0.91	<0.001
mRS 1	-0.05 (-0.08 to -0.02)	0.85	-0.06 (-0.12 to 0.00)	0.83	
mRS 2	-0.16 (-0.21 to -0.12)	0.73	-0.16 (-0.22 to -0.10)	0.73	
mRS 3	-0.27 (-0.32 to -0.23)	0.62	-0.28 (-0.34 to -0.23)	0.61	
mRS 4	-0.46 (-0.50 to -0.41)	0.44	-0.52 (-0.58 to -0.47)	0.37	
mRS 5	-0.60 (-0.67 to -0.53)	0.29	-0.64 (-0.70 to -0.57)	0.26	
mRS 6	–	0.00	–	0.00	
R^2	0.486		0.594		
RMSE	0.171		0.178		
MAE	0.128		0.137		
Tobit					
Intercept	0.97 (0.93 to 1.00)		0.98 (0.92 to 1.05)		
mRS 0	Reference	0.97	Reference	0.98	0.008
mRS 1	-0.08 (-0.12 to -0.03)	0.82	-0.10 (-0.17 to -0.03)	0.80	
mRS 2	-0.22 (-0.28 to -0.16)	0.68	-0.22 (-0.29 to -0.14)	0.68	
mRS 3	-0.33 (-0.39 to -0.28)	0.56	-0.35 (-0.42 to -0.28)	0.54	
mRS 4	-0.52 (-0.58 to -0.47)	0.37	-0.59 (-0.66 to -0.53)	0.30	
mRS 5	-0.67 (-0.75 to -0.58)	0.23	-0.71 (-0.78 to -0.63)	0.19	
mRS 6	–	0.00	–	0.00	
R^2	0.457		0.586		
RMSE	0.176		0.180		
MAE	0.130		0.137		

mRS: modified Rankin Scale score, B: partial regression coefficient, UW-mRS: utility-weighted mRS, $P_{interaction}$: P for interaction, OLS: ordinary least squares, R^2: adjusted R-square, RMSE: root mean square error, MAE: mean absolute error.

Patients were stratified into two groups according to the time from onset to EQ-5D-5L assessment (≤14 days and >14 days). OLS regression and Tobit regression were used to estimate the intercept and partial regression coefficients for each mRS score. Point estimates are presented with their 95% confidence intervals. UW-mRS values were derived from the point estimates for each mRS score. The P value for interaction was evaluated by adding an interaction term of mRS × the time from onset to EQ-5D-5L assessment in the model.

Table S8. Association between mRS and EQ-5D utility values stratified by respondents

	Self-reported		Proxy-reported		
	B	UW-mRS	B	UW-mRS	$P_{interaction}$
OLS					
Intercept	0.90 (0.88 to 0.93)		0.75 (0.60 to 0.89)		
mRS 0	Reference	0.90	Reference	0.75	0.84
mRS 1	-0.05 (-0.08 to -0.03)	0.85	-0.07 (-0.27 to 0.13)	0.68	
mRS 2	-0.15 (-0.19 to -0.12)	0.75	-0.23 (-0.40 to -0.05)	0.52	
mRS 3	-0.26 (-0.30 to -0.23)	0.64	-0.21 (-0.36 to -0.05)	0.54	
mRS 4	-0.47 (-0.50 to -0.43)	0.44	-0.42 (-0.57 to -0.27)	0.33	
mRS 5	-0.48 (-0.55 to -0.41)	0.42	-0.51 (-0.66 to -0.36)	0.24	
mRS 6	–	0.00	–	0.00	
R^2	0.457		0.446		
RMSE	0.174		0.147		
MAE	0.132		0.118		
Tobit					
Intercept	0.98 (0.94 to 1.01)		0.76 (0.61 to 0.91)		
mRS 0	Reference	0.98	Reference	0.76	0.35
mRS 1	-0.09 (-0.13 to -0.05)	0.89	-0.08 (-0.28 to 0.11)	0.68	
mRS 2	-0.21 (-0.25 to -0.16)	0.77	-0.24 (-0.42 to -0.06)	0.52	
mRS 3	-0.33 (-0.37 to -0.28)	0.65	-0.22 (-0.38 to -0.07)	0.54	
mRS 4	-0.54 (-0.58 to -0.49)	0.44	-0.43 (-0.58 to -0.28)	0.33	
mRS 5	-0.54 (-0.63 to -0.46)	0.43	-0.52 (-0.67 to -0.37)	0.24	
mRS 6	–	0.00	–	0.00	
R^2	0.431		0.446		
RMSE	0.178		0.147		
MAE	0.133		0.118		

mRS: modified Rankin Scale score, B: partial regression coefficient, UW-mRS: utility-weighted mRS, $P_{interaction}$: P for interaction, OLS: ordinary least squares, R^2: adjusted R-square, RMSE: root mean square error, MAE: mean absolute error.

Patients were stratified into two groups according to the respondents (self-reported and proxy-reported). OLS regression and Tobit regression were used to estimate the intercept and partial regression coefficients for each mRS score. Point estimates are presented with their 95% confidence intervals. UW-mRS values were derived from the point estimates for each mRS score. The P value for interaction was evaluated by adding an interaction term of mRS × the respondents in the model.

Table S9. Background characteristics of included and excluded patients

	Included	Excluded	P
Age, y	75 (67–83)	81 (73–87)	<0.001
Female	596 (41.0)	115 (52.3)	0.002
Body mass index, kg/m^2	22. 8 (20. 5–25.2)	21.9 (19.6–24.2)	0.002
Risk factors			
Hypertension	1217 (83.8)	182 (82.7)	0.76
Diabetes mellitus	436 (30.0)	64 (29.1)	0.84
Dyslipidemia	893 (61.5)	102 (46.4)	<0.001
Atrial fibrillation	354 (24.4)	80 (36.4)	<0.001
Smoking	782 (53.9)	95 (43.2)	0.004
Drinking	498 (34.3)	50 (22.7)	0.001
Previous stroke	281 (19.4)	43 (19.5)	<0.001
Pre-stroke dependency	492 (33.9)	120 (54.5)	<0.001
Stroke subtype			
Cardioembolism	327 (22.5)	75 (34.1)	<0.001
Non-cardioembolism			
Large-artery atherosclerosis	210 (14.5)	34 (15.5)	
Small-vessel occlusion	518 (35.7)	50 (22.7)	
Others	397 (27.3)	61 (27.7)	
NIHSS score	1 (0–3)	5 (1–13)	<0.001
mRS score	2 (1–4)	4 (2–5)	<0.001

NIHSS: National Institutes of Health Stroke Scale, mRS: modified Rankin Scale.
Data are presented as median (interquartile range) or n (%).

Table S10. Association between mRS and EQ-5D utility values before and after multiple imputation

	Before imputation		After imputation	
	B	UW-mRS	B	UW-mRS
OLS				
Intercept	0.90 (0.88 to 0.92)		0.90 (0.87 to 0.92)	
mRS 0	Reference	0.90	Reference	0.90
mRS 1	-0.05 (-0.08 to -0.02)	0.85	-0.05 (-0.08 to -0.02)	0.85
mRS 2	-0.16 (-0.19 to -0.12)	0.74	-0.16 (-0.19 to -0.12)	0.74
mRS 3	-0.28 (-0.31 to -0.24)	0.62	-0.28 (-0.31 to -0.24)	0.62
mRS 4	-0.49 (-0.53 to -0.46)	0.41	-0.50 (-0.54 to -0.47)	0.40
mRS 5	-0.62 (-0.66 to -0.58)	0.28	-0.62 (-0.66 to -0.58)	0.28
mRS 6	–	0.00	–	0.00
R^2	0.577		0.603	
RMSE	0.174		0.176	
MAE	0.132		0.133	
Tobit				
Intercept	0.97 (0.94 to 1.00)		0.97 (0.94 to 1.00)	
mRS 0	Reference	0.97	Reference	0.97
mRS 1	-0.08 (-0.12 to -0.05)	0.89	-0.08 (-0.12 to -0.04)	0.89
mRS 2	-0.21 (-0.26 to -0.17)	0.76	-0.21 (-0.25 to -0.17)	0.76
mRS 3	-0.34 (-0.38 to -0.30)	0.63	-0.34 (-0.38 to -0.30)	0.63
mRS 4	-0.56 (-0.60 to -0.52)	0.41	-0.57 (-0.61 to -0.53)	0.40
mRS 5	-0.69 (-0.74 to -0.64)	0.28	-0.69 (-0.73 to -0.64)	0.28
mRS 6	–	0.00	–	0.00
R^2	0.562		0.601	
RMSE	0.178		0.178	
MAE	0.133		0.134	

mRS: modified Rankin Scale score, B: partial regression coefficient, UW-mRS: utility-weighted mRS, OLS: ordinary least squares, R^2: adjusted R-square, RMSE: root mean square error, MAE: mean absolute error.
During the study period, 220 patients had incomplete responses or missing data for the EQ-5D. Missing utility values for these patients were imputed using multiple imputation. OLS regression and Tobit regression were used to estimate the intercept and partial regression coefficients for each mRS score. Point estimates are presented with their 95% confidence intervals. UW-mRS values were derived from the point estimates for each mRS score.

Table S11. Association between mRS and EQ-5D utility values after excluding patients with pre-stroke dementia, Parkinsonism, or depression

	OLS		Tobit	
	B	UW-mRS	B	UW-mRS
Intercept	0.90 (0.87 to 0.92)		0.96 (0.93 to 1.00)	
mRS 0	Reference	0.90	Reference	0.96
mRS 1	-0.05 (-0.08 to -0.02)	0.85	-0.08 (-0.11 to -0.04)	0.82
mRS 2	-0.15 (-0.18 to -0.11)	0.75	-0.20 (-0.24 to -0.16)	0.70
mRS 3	-0.28 (-0.31 to -0.24)	0.62	-0.34 (-0.38 to -0.30)	0.56
mRS 4	-0.49 (-0.52 to -0.45)	0.41	-0.55 (-0.59 to -0.51)	0.35
mRS 5	-0.62 (-0.67 to -0.58)	0.28	-0.69 (-0.74 to -0.63)	0.21
mRS 6	–	0.00	–	0.00
R^2	0.571		0.554	
RMSE	0.171		0.174	
MAE	0.130		0.131	

mRS: modified Rankin Scale score, OLS: ordinary least squares, B: partial regression coefficient, UW-mRS: utility-weighted mRS, R^2: adjusted R-square, RMSE: root mean square error, MAE: mean absolute error.

As a sensitivity analysis, 171 patients with pre-stroke dementia, Parkinsonism, or depression were excluded from the analysis. OLS regression and Tobit regression were used to estimate the intercept and partial regression coefficients for each mRS score. Point estimates are presented with their 95% confidence intervals. UW-mRS values were derived from the point estimates for each mRS score.

Appendix

Participating Hospitals

The hospitals participating in the Fukuoka Stroke Registry included Kyushu University Hospital (Fukuoka, Japan), National Hospital Organization Kyushu Medical Center (Fukuoka, Japan), National Hospital Organization Fukuoka-Higashi Medical Center (Koga, Japan), Fukuoka Red Cross Hospital (Fukuoka, Japan), St. Mary's Hospital (Kurume, Japan), Steel Memorial Yawata Hospital (Kitakyushu, Japan), and Japan Labor Health and Welfare Organization Kyushu Rosai Hospital (Kitakyushu, Japan).

Fukuoka Stroke Registry Investigators

Steering committee and research working group members of the Fukuoka Stroke Registry were Takao Ishitsuka, MD, PhD (Seiai Rehabilitation Hospital, Onojo, Japan); Setsuro Ibayashi, MD, PhD (Chair, Seiai Rehabilitation Hospital, Onojo, Japan); Kenji Kusuda, MD, PhD (Seiai Rehabilitation Hospital, Onojo, Japan); Kenichiro Fujii, MD, PhD (Japan Seafarers Relief Association Moji Ekisaikai Hospital, Kitakyushu, Japan); Tetsuhiko Nagao, MD, PhD (Safety Monitoring Committee, Seiai Rehabilitation Hospital, Onojo, Japan); Yasushi Okada, MD, PhD (Vice-Chair, National Hospital Organization Kyushu Medical Center, Fukuoka, Japan); Masahiro Yasaka, MD, PhD (Fukuoka Neurosurgical Hospital, Fukuoka, Japan); Hiroaki Ooboshi, MD, PhD (Seiai Rehabilitation Hospital, Onojo, Japan); Takanari Kitazono, MD, PhD (Principal Investigator, Kyushu University, Fukuoka, Japan); Katsumi Irie, MD, PhD (Hakujyuji Hospital, Fukuoka, Japan); Tsuyoshi Omae, MD, PhD (Imazu Red Cross Hospital, Fukuoka, Japan); Kazunori Toyoda, MD, PhD (National Cerebral and Cardiovascular Center, Suita, Japan); Hiroshi Nakane, MD, PhD (National Hospital Organization Fukuoka-Higashi Medical Center, Koga, Japan); Masahiro Kamouchi, MD, PhD (Kyushu University, Fukuoka, Japan); Hiroshi Sugimori, MD, PhD (National Hospital Organization Kyushu Medical Center, Fukuoka, Japan); Shuji Arakawa, MD, PhD (Steel Memorial Yawata Hospital, Kitakyushu, Japan); Kenji Fukuda, MD, PhD (St Mary's Hospital, Kurume, Japan); Tetsuro Ago, MD, PhD (Kyushu University, Fukuoka, Japan); Jiro Kitayama, MD, PhD (Fukuoka Red Cross Hospital, Fukuoka, Japan); Shigeru Fujimoto, MD, PhD (Jichi Medical University, Shimotsuke, Japan); Shoji Arihiro, MD (Japan Labor Health and Welfare Organization Kyushu Rosai Hospital, Kitakyushu, Japan); Junya Kuroda, MD, PhD (National Hospital Organization Fukuoka-Higashi Medical Center, Koga, Japan); Yoshinobu Wakisaka, MD, PhD (Kyushu University Hospital, Fukuoka, Japan); Yoshihisa Fukushima, MD (St Mary's Hospital, Kurume, Japan); Ryu Matsuo, MD, PhD (Secretariat, Kyushu University, Fukuoka, Japan); Fumi Irie, MD, PhD (Kyushu University, Fukuoka, Japan); Kuniyuki Nakamura, MD, PhD (Kyushu University Hospital, Fukuoka, Japan); and Takuya Kiyohara, MD, PhD (Kyushu University Hospital, Fukuoka, Japan).
